# Erratum

**DOI:** 10.1590/1980-549720220006.supl.2erratum

**Published:** 2024-09-30

In the manuscript "**Sociodemographic and residential factors associated with multimorbity: results of Brumadinho Health Project**", DOI: https://doi.org/10.1590/1980-549720220006.supl.2, published in the Rev Bras Epidemiol. 2022; 25:e220006.supl.2

**On page 1 it was included:**

SCIENTIFIC EDITOR: Antonio Fernando Boing http://orcid.org/0000-0001-9331-1550

